# Speeding up research with the Semantic Web

**DOI:** 10.1186/1750-1172-7-S2-A11

**Published:** 2012-11-22

**Authors:** Marco Roos, Erik A Schultes, Barend Mons

**Affiliations:** 1 Human Genetics Department, Leiden University Medical Center, Leiden, 2333 ZA, The Netherlands

## 

Data for Rare Diseases are often distributed. Ideally, we can combine relevant data and biological insights from any place in the world and use it directly as input for computational analysis. However, too often data is poorly described making it hard to find, hard to assess its quality, and hard to integrate with other data. A valid question is: 'Why can't we analyse data as if it came from one global database?'. Here we introduce the Semantic Web as an enabling technology for making data interoperable and thereby expediting biological insight.

The Semantic Web 'language' is RDF: the Resource Description Framework. It uses the 'hyperlink' mechanism known from the internet to refer to data instead of web pages. Meaningful relations are specified as triples: subject, predicate, object. For example, 'CAPN3', *'interacts with'*, 'ParvB'. Written in RDF:

http://www.uniprot.org/uniprot/P20807

http://www.conceptwiki.org/index.php/Concept:adf6044e-5c2b-11df-b0cb-001517ac506c

http://bio2rdf.org/geneid:29780

While RDF is meant for computers, we see that: (i) RDF triples convey meaning; (ii) hyperlinks specify the location of data, which might be different databases (even within a triple); (iii) data items are also references to other RDF documents with more triples (e.g. try http://www.uniprot.org/uniprot/Q13547 in a browser). A hyperlink can be in any number of triples, effectively creating the world wide database of meaningfully linked data that is needed in the study of Rare Diseases. Ontologies can also be encoded in RDF, thereby extending the functionality to a global knowledge base. New experiments and discoveries can continually add information to this knowledge base.

For example, the Semantic Web can help us to find drug targets for Rare Diseases. For this purpose, OpenPhacts [1] is integrating compounds from Chemspider [http://chemspider.com], proteins from UniProt [http://uniprot.org], pathways from WikiPathways [http://wikipathways.org], and documents from PubMed [http://www.ncbi.nlm.nih.gov/pubmed/]. We also make DNA sequence variations from the Leiden Open Variation Database (LOVD [http://www.lovd.nl]) available in RDF, and visualised via the UCSC genome browser.

However, a number of barriers must be overcome. First, databases pre-dating the Semantic Web are used abundantly and must be integrated. This is usually an expensive and tedious task. Secondly, building a scientific reputation often conflicts with data sharing. Therefore, we have developed a data publishing framework called Nanopublication: an application of RDF that links authorship to individual datum (attribution). This creates a transparent and equitable incentive for data sharing. Nano publications also provide incentives for the exposure of legacy data.

In conclusion, Nano publications and Semantic Web technology makes data easier to find and directly applicable to integrative analyses.
